# *In Vitro* Reconstitution Defines the Minimal Requirements for Cdc48-Dependent Disassembly of the CMG Helicase in Budding Yeast

**DOI:** 10.1016/j.celrep.2019.08.026

**Published:** 2019-09-10

**Authors:** Progya P. Mukherjee, Karim P.M. Labib

**Affiliations:** 1 The MRC Protein Phosphorylation and Ubiquitylation Unit, School of Life Sciences, University of Dundee, Dundee, Scotland DD1 5EH, UK

**Keywords:** DNA replication, CMG helicase, ubiquitylation, Mcm7, SCF^Dia2^, Cdc48, ATPase, p97, Ufd1-Npl4

## Abstract

Disassembly of the replisome is the final step of chromosome duplication in eukaryotes. In budding yeast and metazoa, cullin ubiquitin ligases are required to ubiquitylate the Cdc45-MCM-GINS (CMG) helicase that lies at the heart of the replisome, leading to a disassembly reaction that is dependent upon the ATPase known as Cdc48 or p97. Here, we describe the reconstitution of replisome disassembly, using a purified complex of the budding yeast replisome in association with the cullin ligase SCF^Dia2^. Upon addition of E1 and E2 enzymes, together with ubiquitin and ATP, the CMG helicase is ubiquitylated on its Mcm7 subunit. Subsequent addition of Cdc48, together with its cofactors Ufd1-Npl4, drives efficient disassembly of ubiquitylated CMG, thereby recapitulating the steps of replisome disassembly that are observed *in vivo*. Our findings define the minimal requirements for disassembly of the eukaryotic replisome and provide a model system for studying the disassembly of protein complexes by Cdc48-Ufd1-Npl4.

## Highlights

• *In vitro* ubiquitylation of budding yeast replisome by SCF^Dia2^
• SCF^Dia2^ ubiquitylates the CMG helicase without priming by HECT or RBR ligases
• *In vitro* reconstitution of the disassembly of ubiquitylated CMG
• Budding yeast Cdc48-Ufd1-Npl4 are sufficient to disassemble ubiquitylated CMG

## Graphical Abstract

**Figure:**
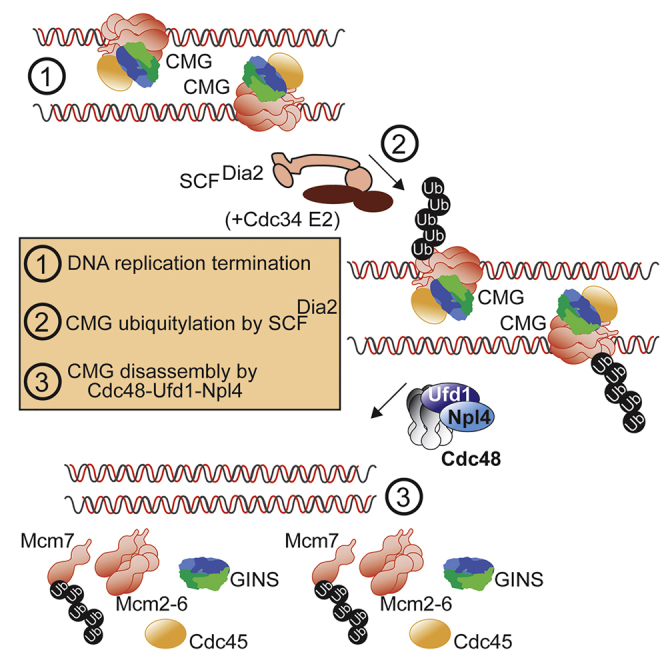

## Teaser

To study the mechanism of CMG helicase disassembly during DNA replication termination, Mukherjee and Labib purify a complex of the yeast replisome with the E3 ligase SCF^Dia2^. After *in vitro* ubiquitylation of the Mcm7 subunit of CMG, recombinant Cdc48 with its adaptors Ufd1-Npl4 are sufficient to drive efficient CMG disassembly.

## Introduction

During the initiation of chromosome duplication in eukaryotes, the replisome assembles at nascent DNA replication forks around the 11-subunit Cdc45-MCM-GINS (CMG) helicase, which then associates tightly with DNA replication forks throughout elongation via the hexameric Mcm2-7 motor of CMG that encircles the template of the leading strand (Bell and Labib, 2016, Burgers and Kunkel, 2017, Deegan and Diffley, 2016). During DNA replication termination, the Mcm7 subunit of CMG is ubiquitylated and the replisome disassembled (Maric et al., 2014, Moreno et al., 2014), in a poorly characterized reaction that has not previously been reconstituted with purified proteins (Dewar and Walter, 2017, Gambus, 2017). Ubiquitylation of Mcm7 during termination requires the cullin E3 ligase SCF^Dia2^ in budding yeast (Maric et al., 2014, Maric et al., 2017) and a distinct cullin ligase known as CUL2^LRR1^ in metazoa (Dewar et al., 2017, Sonneville et al., 2017). At present, however, it remains unclear whether other factors also contribute to Mcm7 ubiquitylation during termination, such as other E3 ligases that are thought to prime the ubiquitylation of cullin ligase substrates (Dove et al., 2017, Harreman et al., 2009, Kelsall et al., 2019, Scott et al., 2016). The TRAIP ubiquitin ligase promotes CMG ubiquitylation in metazoa, but TRAIP does not function during DNA replication termination and instead acts in distinct pathways for CMG disassembly that are restricted to mitosis or certain forms of DNA damage (Deng et al., 2019, Wu et al., 2019).

Past studies found that disassembly of ubiquitylated CMG can be inhibited by inactivation of the Cdc48 AAA+ ATPase (Dewar et al., 2015, Maric et al., 2014, Moreno et al., 2014), which is able to disrupt highly stable protein complexes after their ubiquitylation (Bodnar and Rapoport, 2017a, Franz et al., 2016, van den Boom et al., 2016, Vaz et al., 2013), though such reactions have yet to be reconstituted with purified proteins. CMG disassembly also requires the Ufd1-Npl4 adaptors of Cdc48 (Franz et al., 2011, Maric et al., 2017, Sonneville et al., 2017), which bind to ubiquitin chains and help to recruit Cdc48 to ubiquitylated substrates (Bodnar et al., 2018). In order to explore the mechanism of replisome disassembly during DNA replication termination, it is first necessary to reconstitute the process with purified components and thereby establish the minimal requirements for disassembly of a protein complex by the Cdc48 segregase.

## Results

### RBR Ligases, HECT Ligases, and Cullin Neddylation Are Dispensable for CMG Ubiquitylation in Budding Yeast

**Figure 1:**
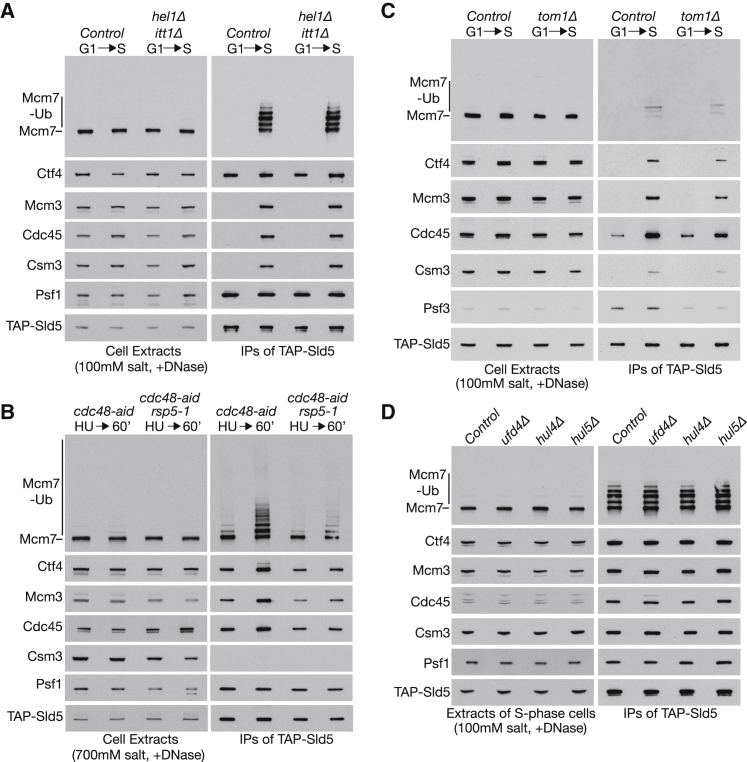
RBR and HECT E3 Ligases Are Dispensable for CMG Ubiquitylation in Budding Yeast (A) *TAP-SLD5* control cells (YSS47) and *TAP-SLD5 hel1Δ itt1Δ* cells (YPM141) were grown at 24°C and synchronized in G1 phase by addition of mating pheromone before release into S phase for 30 min. Samples were used to make “low-salt” cell extracts (100 mM KOAc), in which *in vitro* CMG ubiquitylation was monitored by immunoprecipitation of the TAP-tagged Sld5 subunit of GINS. The indicated proteins were monitored by immunoblotting. DNA content was also monitored by flow cytometry (see Figure S1A). (B) *TAP-SLD5* (YMM228) and *TAP-SLD5 rsp5-1* (YPM174) cells were synchronized in G1 phase at 24°C and then released into fresh medium containing 0.2M hydroxyurea (HU) to arrest cells in early S phase. The cultures were then shifted to 37°C for 60 min in the presence of 500 μM indoleacetic acid (auxin) in order to inactivate Rsp5-1 and deplete Cdc48-aid. The cells were subsequently released for 60 min at 37°C into fresh medium lacking HU but containing auxin. “High-salt” extracts were made in the presence of 700 mM KOAc in order to inhibit *in vitro* CMG ubiquitylation (Maric et al., 2014) and thus reveal *in vivo* ubiquitylation. After digestion of chromosomal DNA, TAP-Sld5 was isolated by immunoprecipitation and the associated factors monitored as above. (C) *TAP-SLD5* (YSS47) and *TAP-SLD5 tom1Δ* (YPM44) cells were grown and processed as in (A). (D) *TAP-SLD5* (YSS47), *TAP-SLD5 ufd4Δ* (YPM150), *TAP-SLD5 hul4Δ* (YPM192), and *TAP-SLD5 hul5Δ* (YPM195) cells were treated as above (in this case only the S phase samples are shown). See also Figure S1.

Recent studies of substrate ubiquitylation by cullin ubiquitin ligases in metazoa indicate that the reaction can be surprisingly complex, with addition of the first ubiquitin being driven by a member of the Ariadne family of RBR (ring between ring) ubiquitin ligases, before the cullin ligase then promotes subsequent polyubiquitylation (Dove et al., 2017, Scott et al., 2016). Budding yeast lacks orthologs of Ariadne but has two RBR ligases known as Hel1 and Itt1. To test whether these enzymes are important for ubiquitylation of the Mcm7 subunit of CMG, we synchronized cells in S phase and prepared “low-salt” extracts (see STAR Methods), in which the endogenous CMG helicase from replication forks is ubiquitylated *in vitro*, dependent upon SCF^Dia2^ (Maric et al., 2014). As shown in Figures 1A and S1A, ubiquitylation of the Mcm7 subunit of CMG was comparable in extracts of control cells and in cells lacking both Hel1 and Itt1. Therefore, RBR ligases are not required for SCF^Dia2^-dependent CMG ubiquitylation in budding yeast.

Previous work showed that polyubiquitylation of RNA polymerase in budding yeast is dependent not only on the cullin ligase Cul3 but also on a HECT (homologous to the E6-AP carboxyl terminus) ligase known as Rsp5 (Harreman et al., 2009). To test whether Rsp5 might also be required for CMG ubiquitylation by SCF^Dia2^, perhaps acting as a priming enzyme, we monitored *in vivo* ubiquitylation of CMG when *cdc48-aid* cells or *cdc48-aid rsp5-1* cells progressed synchronously through S phase at the restrictive temperature of 37°C, following depletion of degron-tagged Cdc48-aid to prevent disassembly of ubiquitylated CMG helicase (Figure S1B; “high-salt” extracts were used to prevent *in vitro* CMG ubiquitylation, and inactivation of Cdc48-aid prevented disassembly of *in vivo* ubiquitylated CMG). As shown previously (Maric et al., 2014), *in vivo* CMG ubiquitylation under such conditions is dependent upon SCF^Dia2^. Note that Rsp5 is essential for viability, and so we used the temperature-sensitive allele *rsp5-1* and monitored *in vivo* ubiquitylation during DNA replication termination at 37°C in order to avoid the potential reactivation of the Rsp5-1 protein in cell extracts during *in vitro* assays.

As shown in Figure 1B, the CMG helicase accumulated with ubiquitylated Mcm7 subunit upon release of *cdc48-aid* cells from early S phase arrest after inactivation of Cdc48-aid (Figure 1B; *cdc48-aid*; 60’). Similarly, Mcm7 ubiquitylation was also detected when *cdc48-aid rsp5-1* cells were released from S phase arrest (Figure 1B). In comparison with control cells released from early S phase arrest, the *cdc48-aid rsp5-1* mutant contained less ubiquitylated Mcm7 but also contained correspondingly less CMG (compare Mcm7, Mcm3, Cdc45, and the GINS subunits Sld5 and Psf1 in the immunoprecipitates of TAP-Sld5 from both strains). This suggested that the activation of late origins of replication might be impaired in *rsp5-1* cells under these conditions but indicated that Rps5 is not essential for CMG ubiquitylation in budding yeast cells.

To test whether any of the other HECT ligases in budding yeast might play a role in CMG ubiquitylation, we compared *in vitro* ubiquitylation of the Mcm7 subunit of CMG in extracts of control cells or cells lacking the Tom1, Ufd4, Hul4, or Hul5 HECT ligases (Figures 1C, 1D, S1C, and S1D). In each case, CMG ubiquitylation was comparable to control cell extracts. Overall, therefore, these data indicate that CMG ubiquitylation in budding yeast, which is dependent upon SCF^Dia2^, does not additionally require RBR ligases or any one of the various HECT ubiquitin ligases.

In vertebrates, cullin neddylation is essential for the function of Cullin ubiquitin ligases (Boh et al., 2011, Duda et al., 2008, Saha and Deshaies, 2008). Indeed, previous work showed that neddylation of Cul2 is essential for CMG ubiquitylation and disassembly in extracts of *Xenopus laevis* eggs (Dewar et al., 2017, Moreno et al., 2014, Sonneville et al., 2017). Cullin neddylation is conserved in yeasts, but the Rub1 ortholog of NEDD8 in budding yeast is dispensable for cell viability (Lammer et al., 1998, Liakopoulos et al., 1998). Correspondingly, we found that CMG ubiquitylation in *rub1Δ* cells was modestly reduced, but not abolished, both in yeast cell extracts (Figures S2A and S2B) and also *in vivo* (Figures S2C and S2D). These findings suggested that it should be possible to reconstitute CMG ubiquitylation by SCF^Dia2^ without requiring either cullin neddylation or other ubiquitin ligases.

### Optimizing Conditions for CMG Ubiquitylation in Yeast Cell Extracts

**Figure 2:**
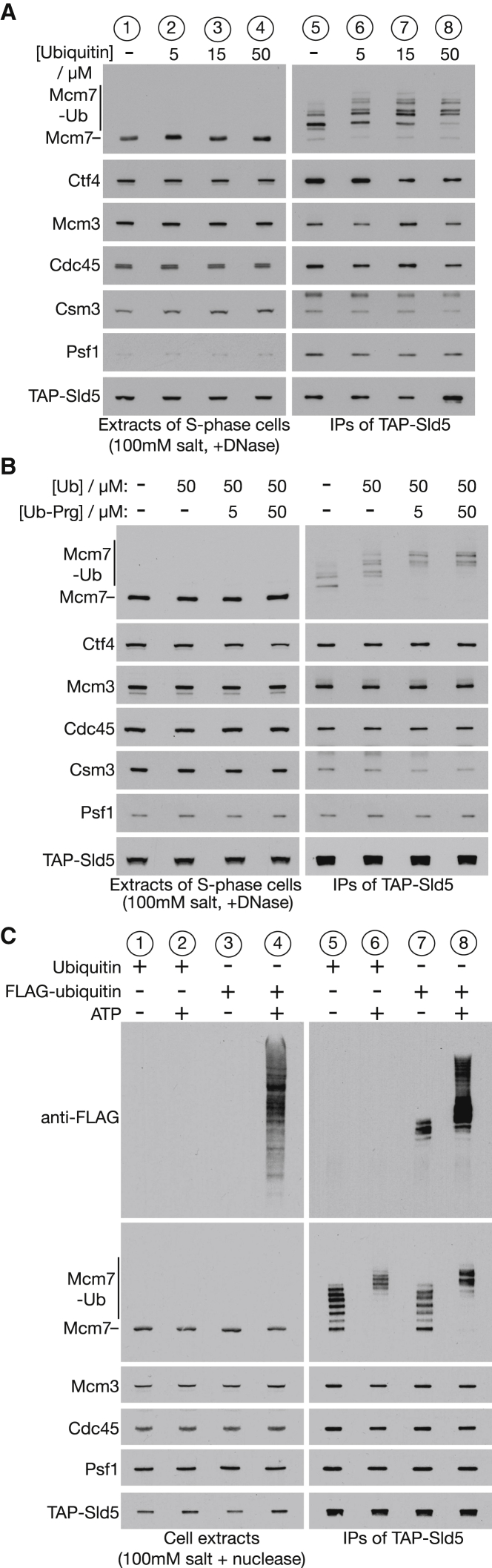
Optimization of CMG Ubiquitylation in Yeast Cell Extracts (A) *TAP-SLD5* cells (YSS47) were synchronized in early S phase and used to prepare cell extracts as in Figure 1A. Recombinant ubiquitin was added at the indicated concentrations, before digestion of chromosomal DNA and isolation of the CMG helicase by immunoprecipitation of the TAP-tagged Sld5 subunit of GINS. The indicated proteins were then monitored by immunoblotting. (B) In a similar experiment, wild-type ubiquitin (Ub) or the deubiquitylase inhibitor propargylated ubiquitin (Ub-Prg) were added to cell extracts at the indicated concentrations. (C) In a similar experiment, 50 μM wild-type ubiquitin, 50 μM FLAG-tagged ubiquitin, and 3 mM ATP were added as indicated to an extract of S phase cells, together with 5 μM Ubi-Prg. See also Figures S1 and S2.

Before attempting to reconstitute CMG ubiquitylation with purified proteins, we first investigated further the *in vitro* ubiquitylation of CMG in yeast cell extracts (Maric et al., 2014). Though CMG ubiquitylation is normally restricted to DNA replication termination, almost all of the CMG helicase in S phase cells can be ubiquitylated *in vitro*, when high pH extracts are prepared in the presence of nuclease (Maric et al., 2014) in order to release CMG from DNA replication forks and thus facilitate its subsequent purification. K48-linked ubiquitin chains are conjugated to Mcm7 under such conditions (Maric et al., 2014), but the chains are short (Figure 2A, lane 5—the strongest band corresponds to conjugation of two ubiquitins to Mcm7), and the ubiquitylated CMG helicase is not disassembled in the cell extracts.

To investigate whether CMG ubiquitylation can be improved further under such conditions, we added recombinant ubiquitin to the extract and found that several additional ubiquitins were conjugated to Mcm7 (Figure 2A, lanes 6–8; up to 6 or 7 ubiquitins were conjugated to Mcm7). Because deubiquitylases (DUBs) in the cell extract might also limit CMG ubiquitylation, we inhibited cysteine DUBs by addition of propargylated ubiquitin (Ekkebus et al., 2013) and observed a further enhancement of CMG ubiquitylation in combination with recombinant wild-type ubiquitin, such that up to 8 ubiquitin moieties were conjugated to Mcm7 (Figure 2B; Ub-Prg).

Finally, we tested whether the concentration of ATP in the extract was limiting for CMG ubiquitylation. Addition of 3 mM ATP led to a dramatic stimulation of bulk ubiquitylation in the cell extract, as revealed by addition of FLAG-tagged recombinant ubiquitin (Figure 2C, lane 4). In combination with 5 μM Ubi-Prg and 50 μM wild-type ubiquitin (either untagged or FLAG tagged), we found that 3 mM ATP stimulated CMG ubiquitylation, such that up to 10 ubiquitins were conjugated to Mcm7 (Figure 2C, lanes 6 and 8). However, this still did not lead reproducibly to CMG disassembly under these conditions. Therefore, we next attempted to establish a reconstituted system based on a partially purified substrate in order to optimize the reaction further.

### A Purified Complex of SCF^Dia2^ and the Budding Yeast Replisome Supports Mcm7 Ubiquitylation *In Vitro*

**Figure 3:**
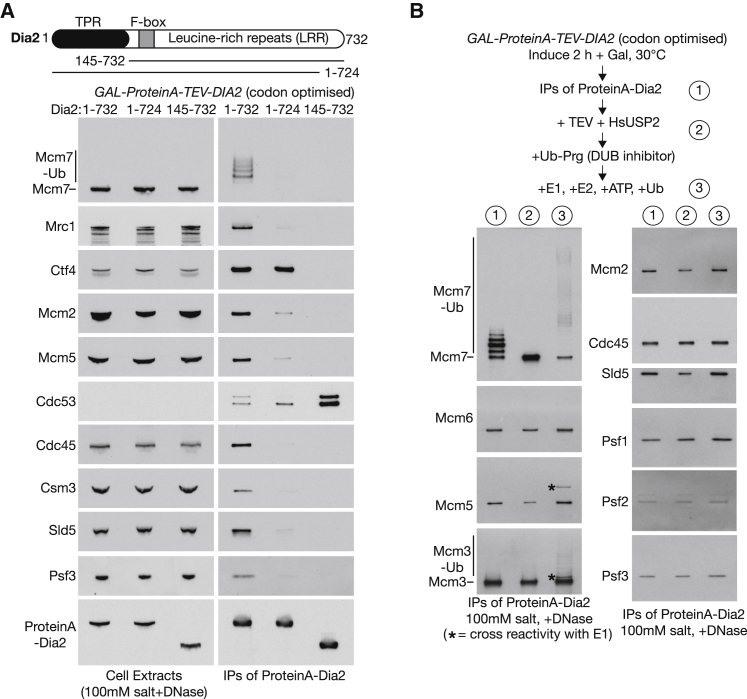
A Complex of SCF^Dia2^ with the Replisome Supports *In Vitro* Ubiquitylation of the CMG Helicase by Cdc34 (A) Asynchronous cultures of cells with ProteinA-tagged versions of Dia2 1–732 (full-length Dia2; YTM418), Dia2 1–724 (deletion of Dia2 TPR domain; YPM220), or Dia2 145–732 (small truncation in Dia2 LRR domain; YTM495) under the control of the *GAL1,10* promoter were grown at 30°C in medium containing raffinose before induction for 2 h in medium containing galactose. The tagged proteins were then isolated from cell extracts by immunoprecipitation on IgG-coupled beads. Half of each sample was treated with TEV protease to release bound proteins from the beads (to avoid interference of TAP-tagged protein in immunoblots of similarly sized proteins), whereas the other half was analyzed by boiling directly in Laemmli buffer before SDS-PAGE. (B) ProteinA-tagged full-length Dia2 (from YTM418) was isolated as above (sample 1) before release from beads with TEV protease and deubiquitylation with HsUSP2 for 1 h at 24°C (sample 2). After treatment for 15 min with the DUB inhibitor Ubi-Prg, samples were treated for a further 30 min at 24°C with 30 nM Uba1 E1 enzymes, 150 nM Cdc34 E2 enzyme, 50 μM ubiquitin, and 2.5 mM ATP. The indicated proteins were then monitored by immunoblotting. See also Figures S1, S3, and S4.

We previously showed that SCF^Dia2^ can associate with the yeast replisome (Morohashi et al., 2009), dependent upon partners of the CMG helicase, such as Ctf4 and Mrc1, which are important for efficient ubiquitylation of Mcm7 in yeast cell extracts (Maculins et al., 2015). In order to generate a substrate for *in vitro* ubiquitylation of CMG in the context of the replisome, we overexpressed a codon-optimized version of ProteinA-tagged Dia2 in budding yeast cells and isolated a complex of SCF^Dia2^-replisome on IgG-coated magnetic beads (Figure 3). As shown in Figure 3A, the association of SCF^Dia2^ with the yeast replisome was dependent upon the amino terminal tetratricopeptide repeat (TPR) domain of Dia2 that interacts with Ctf4 and Mrc1, consistent with previous findings (Morohashi et al., 2009). Interestingly, however, the association of SCF^Dia2^ with the replisome was also impaired by a small C-terminal truncation of the leucine-rich repeats (LRRs) domain, which is thought to represent the substrate binding module of the E3 ligase SCF^Dia2^. These findings indicate that SCF^Dia2^ is recruited to the replisome via multiple interactions, including with partners of CMG, such as Ctf4 and Mrc1, but probably also involving a direct interaction with CMG itself (likely via binding of the LRR of Dia2 to Mcm7, though this remains to be shown directly).

To generate an *in vitro* assay for CMG ubiquitylation, we released the SCF^Dia2^-replisome complex from the beads by tobacco etch virus (TEV) cleavage of the ProteinA tag on Dia2 and then treated the released material with the broad specificity deubiquitylase HsUSP2 in order to remove the short ubiquitin chains that were added in the yeast cell extract to the Mcm7 subunit of CMG (Figure 3B, compare steps 1 and 2). Subsequently, Ubi-Prg was added to inactivate the HsUSP2 deubiquitylase, and the resultant material was incubated with the Uba1 E1 enzyme, the Cdc34 E2 enzyme for cullin ligases, ubiquitin, and ATP (Figure 3B, step 3; the purified proteins are shown in Figure S3). This led to efficient *in vitro* ubiquitylation of the Mcm7 subunit of the CMG helicase (Figure 3B; note the corresponding reduction of unmodified Mcm7, in parallel to the increase in polyubiquitylated Mcm7). In addition, a low level of Mcm3 ubiquitylation was also detected, probably reflecting the adjacent location of Mcm3 and Mcm7 within the CMG helicase (Costa et al., 2011, Davey et al., 2003, Yuan et al., 2016).

In yeast cell extracts, CMG ubiquitylation is restricted to lysine 29 of Mcm7, whereas other sites on Mcm7 can also be ubiquitylated *in vivo* (Maric et al., 2017). The reconstituted assay reflects the *in vivo* situation, because isolated SCF^Dia2^-replisome complexes from wild-type or *mcm7-K29A* cells were ubiquitylated with comparable efficiency (Figure S4).

### Reconstituted Disassembly of Ubiquitylated CMG Helicase by Cdc48-Ufd1-Npl4

**Figure 4:**
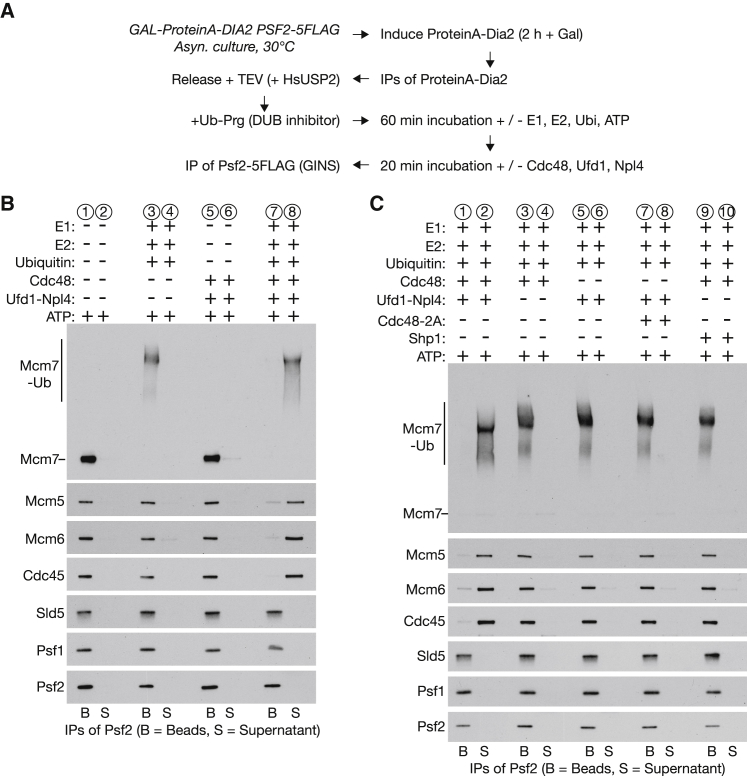
Reconstituted CMG Helicase Disassembly with Purified Proteins Requires Ubiquitylation, the Cdc48 ATPase, and Ufd1-Npl4 (A) *GAL-ProteinA-TEV-DIA2 PSF2-5FLAG* cells (YPM157) were processed as indicated. At the end of the reactions, the GINS component of the CMG helicase was isolated by immunoprecipitation of Sld5, in order to monitor the integrity of CMG. (B) The samples described in (A) were resolved by SDS-PAGE, and the indicated proteins were analyzed by immunoblotting. (C) Similar reactions were performed with the indicated factors. See also Figures S1 and S3.

To establish whether Cdc48, Ufd1, and Npl4 are the only essential factors for replisome disassembly and also test whether *in vitro* ubiquitylated CMG was functional, we developed a reconstituted disassembly assay with purified proteins. We first purified recombinant versions of Cdc48, Ufd1, and Npl4 from *E. coli* (Figure S3), as described previously (Bodnar and Rapoport, 2017b). Subsequently, we isolated unmodified SCF^Dia2^-replisome complexes as above and incubated them for 60 min at 24°C, in the presence or absence of E1-E2-ubiquitin-ATP. Finally, we incubated the complexes for a further 20 min at 24°C, in the presence or absence of Cdc48-Ufd1-Npl4, and then isolated the GINS component of the CMG helicase by immunoprecipitation of the Sld5 subunit (Figure 4A). In this way, we could assay for replisome disassembly by monitoring the presence of replisome proteins on the beads or in the supernatant.

In the absence of ubiquitylation, the integrity of the CMG helicase was unaffected by incubation with ATP at 24°C, regardless of the presence or absence of Cdc48-Ufd1-Npl4 (Figure 4B, compare lanes 1 and 2 and lanes 5 and 6). Moreover, the Mcm7 subunit of CMG was ubiquitylated very efficiently in the presence of E1, E2, and ATP, but this did not lead to CMG disassembly or to the release of CMG components into the supernatant (Figure 4B, lanes 3 and 4). In contrast, Cdc45 and the Mcm2–7 components of CMG were present almost exclusively in the supernatant when SCF^Dia2^-replisome complexes were first ubiquitylated and then incubated with Cdc48-Ufd1-Npl4, before immunoprecipitation of Sld5 (Figure 4B, lane 8), whereas GINS was efficiently retained on the beads (Figure 4B, lane 7). These findings demonstrated that Cdc48-Ufd1-Npl4 are sufficient for CMG helicase disassembly *in vitro*, dependent upon prior ubiquitylation of CMG by SCF^Dia2^ and Cdc34.

In subsequent experiments, we showed that the reconstituted disassembly of ubiquitylated CMG helicase is dependent upon Ufd1-Npl4 (Figure 4C, lanes 3 and 4) and requires not only the presence of Cdc48 protein (Figure 4C, lanes 5 and 6) but also the ATPase activity of the Cdc48 segregase (Figure 4C, lanes 7 and 8; Cdc48-2A has E351A and E588A mutations in the Walker B motifs of the D1 and D2 ATPases of Cdc48). Finally, we purified a recombinant form of the Shp1 adaptor of Cdc48, which also binds ubiquitin and forms complexes with Cdc48 that are mutually exclusive to those containing Ufd1-Npl4 (Schuberth et al., 2004). Strikingly, Shp1 was not able to support the disassembly of ubiquitylated CMG by Cdc48, in contrast to Ufd1-Npl4 (Figure 4C, lanes 9 and 10). These findings indicate that Ufd1-Npl4 play a unique role in replisome disassembly, probably via recruitment of Cdc48 to the K48-linked ubiquitin chains that are conjugated to the Mcm7 subunit of the CMG helicase.

## Discussion

Our data indicate that the minimal requirements for replisome disassembly in eukaryotes are CMG ubiquitylation, followed by disassembly of the ubiquitylated helicase by Cdc48-Ufd1-Npl4. In budding yeast, the cullin E3 ligase SCF^Dia2^ directly promotes CMG ubiquitylation by the E2 enzyme Cdc34, without any requirement for a priming ubiquitylation via an RBR or HECT E3 ligase. SCF^Dia2^ is recruited to the replisome via multiple interactions that are mediated by both the TPR and the LRR domains of the F-box protein Dia2, leading to ubiquitylation at one or more of multiple sites on the Mcm7 subunit of CMG. Although the reaction is normally restricted to DNA replication termination, it is remarkable that the replisome becomes a highly efficient substrate for SCF^Dia2^ upon release from DNA replication forks. It will be important in future studies to reconstitute the ubiquitylation reaction in the context of terminating DNA replication forks, but our findings suggest that the structure of active DNA replication forks, or factors associated with active forks, might normally suppress CMG ubiquitylation during elongation.

The principles of replisome disassembly are likely to be conserved in diverse eukaryotes, despite the fact that Dia2 is only present in yeasts. For example, the cullin ligase CUL2^LRR1^ is required to ubiquitylate the MCM7 subunit of CMG in the nematode *Caenorhabditis elegans* (Sonneville et al., 2017) and the vertebrate *Xenopus laevis* (Dewar et al., 2017, Sonneville et al., 2017), and CDC48-UFD1-NPL4 are then needed to disassemble the ubiquitylated helicase during DNA replication termination (Dewar et al., 2017, Franz et al., 2011, Moreno et al., 2014, Sonneville et al., 2017). Disassembly of ubiquitylated CMG helicase by Cdc48-Ufd1-Npl4 has thus been conserved from fungi to metazoa, though it remains possible that helicase ubiquitylation and disassembly in metazoa might require additional factors not present in yeasts.

The *in vitro* ubiquitylation and disassembly of the CMG helicase provides a model system with which to investigate the mechanism by which Cdc48-Ufd1-Npl4 disassembles ubiquitylated protein complexes. It will be important in subsequent studies to characterize the products of CMG helicase disassembly in order to determine whether the ubiquitylated Mcm7 subunit is unfolded and passed through the central pore of the Cdc48 ATPase and whether any other subunits are also attacked by Cdc48-Ufd1-Npl4.

## STAR★Methods

### Key Resources Table

**Table:** 

REAGENT or RESOURCE	SOURCE	IDENTIFIER
**Antibodies**		

Rabbit “Peroxidase Anti-Peroxidase soluble complex”	Sigma	P1291; RRID:AB_1079562
Sheep anti-Mcm7	Maric et. al, 2014	N/A
Sheep anti-Mcm3	Maric et. al, 2014	N/A
Goat anti-Mcm2	Santa Cruz	sc-9839; RRID:AB_648841
Sheep anti-Mcm5	Maric et. al, 2014	N/A
Sheep anti-Mcm6	Maric et. al, 2014	N/A
Sheep anti-Mrc1	This study	N/A
Sheep anti-Ctf4	Maric et. al, 2014	N/A
Sheep anti-Csm3	Maric et. al, 2014	N/A
Sheep anti-Cdc45	Maric et. al, 2014	N/A
Sheep anti-Psf1	Maric et. al, 2014	N/A
Sheep anti-Psf3	Maric et. al, 2014	N/A
Sheep anti-Sld5	Maric et. al, 2014	N/A
Rabbit anti-FLAG	Sigma-Aldrich	F7425; RRID:AB_439687
Rabbit anti-Cdc53	Santacruz	sc-50444; RRID:AB_1121399
Mouse monoclonal anti-FLAG (M2)	Sigma-Aldrich	F1804; RRID:AB_262044
Mouse monoclonal anti-polyubiquitylated conjugates (FK2)	Enzo Life Sciences	BML-PW8810; RRID:AB_10541840

**Bacterial and Virus Strains**		

*Escherichia coli:* BL21-CodonPlus (DE3)-RIL	Agilent	230245

**Chemicals, Peptides, and Recombinant Proteins**		

Indole-3-acetic acid sodium salt	Sigma-Aldrich	I5148
Ni-NTA agarose	QIAGEN	30210
Roche Complete EDTA-free protease inhibitor cocktail	Roche	000000011873580001
Recombinant human Ubiquitin	MRC PPU Reagents and Services	DU20027
Recombinant Cdc48 (wt and E315A E588A mutant)	This study	N/A
Recombinant Ufd1-Npl4	This study	N/A
Recombinant Shp1	This study	N/A
Recombinant Uba1	This study	N/A
Recombinant Cdc34	This study	N/A
Recombinant FLAG-Ubiquitin	MRC PPU Reagents and Services	DU46789
Recombinant Propargyl-Ubiquitin	MRC PPU Reagents and Services	DU49003
Recombinant K0 human Ubiquitin	MRC PPU Reagents and Services	DU24363
Recombinant human USP2	MRC PPU Reagents and Services	DU13025

**Experimental Models: Organisms/Strains**		

*S. cerevisiae* strains are detailed in Table S1		N/A

**Recombinant DNA**		

Plasmid: 14His-SMT3-Cdc48	Stein et al., 2014	N/A
Plasmid: 14His-SMT3-*cdc48-E315A 588A*	This study	N/A
Plasmid: pRS305-GAL-Mcm7-5FLAG9His	This study	N/A
Plasmid: pRS305-GAL-Mcm7-K29A-5FLAG9His	This study	N/A
Plasmid: pRS306-GAL-ProteinA-3TEV-Dia2(1-724)	This study	N/A

**Software and Algorithms**		

FlowJo	TreeStar Inc.	https://www.flowjo.com

### Lead Contact and Materials Availability

Further information and requests for resources and reagents should be directed to and will be fulfilled by the Lead Contact, Karim Labib (kpmlabib@dundee.ac.uk). The plasmids and yeast strains generated in this study are available upon request.

### Experimental Model and Subject Details

#### Budding yeast, *Saccharomyces* cerevisiae

The yeast strains used in this study are detailed in Table S1 and the Key Resources Table. Cells were grown in YP medium (1% yeast extract, 2% bacteriological peptone) supplemented with 2% glucose (YPD), raffinose (YPRaff) or galactose (YPGal). To synchronize cells in G1-phase, a mid-exponential culture of around 0.7 × 10^7^ cells / ml was treated with 7.5μg/ml of alpha factor mating pheromone (Pepceuticals Limited) for one generation time. Cells were then washed twice with fresh medium, in order to release the culture synchronously into S-phase. To arrest cells in early S-phase, G1-phase cells were released into fresh medium in the presence of 0.2 M Hydroxyurea (Molekula, cat. no. MOLE10872383) and then incubated until 90% of cells had budded.

### Method Details

#### Flow cytometry analysis

For each sample, 1ml of yeast cell culture (approximately 10^7^ cells) was harvested and fixed by resuspension in 1ml of 70% ethanol with vortexing, before storage at 4°C. Subsequently, 150μl of fixed cells were added to 3 mL of 50mM sodium acetate, together with 50μg of RNase A. After incubation at 37°C for 2h, the cells were pelleted and cellular proteins digested at 37°C for 30 minutes by incubation in 500μl of 50mM HCl containing 2.5mg of Pepsin. Finally, cells were pelleted and then re-suspended in 1ml of 50mM Sodium citrate containing 2μg of propidium iodide. Samples were sonicated for 4 s in a Soniprep 150Plus sonicator at 6.9μm amplitude, before analysis in a FACSCanto II flow cytometer (Becton Dickinson), with FlowJo software (TreeStar Inc.).

#### Isolation of replisome from yeast cell extracts by immuno-precipitation of TAP-Sld5

In order to monitor *in vitro* ubiquitylation of the CMG helicase in budding yeast cell extracts, we followed the ‘standard cell extract’ method described previously by Maric et al. (2014), except that Tris-acetate (at pH 9.0) replaced HEPES-KOH (at pH 7.9). The resulting ‘low salt extracts (containing 100 mM potassium acetate) were split into two 1 mL aliquots, each of which was incubated for 2 h at 4°C with 0.95 × 10^8^ magnetic beads (Dynabeads M-270 Epoxy; 14302D, Life Technologies), coupled to rabbit IgG (S1265, Sigma-Aldrich) in order to isolate TAP-Sld5). Subsequently, the beads were washed four times and processed as described previously (Maric et al., 2014).

To monitor *in vivo* ubiquitylation of CMG following inactivation of Cdc48-aid, the procedure was as above, except that ‘high salt’ extracts were prepared by ensuring that all buffers contained 700 mM potassium acetate, since we previously found that *in vitro* ubiquitylation was blocked in the presence of high salt (Maric et al., 2014).

#### Isolation of a complex comprising SCF^Dia2^ and the yeast replisome

ProteinA-tagged Dia2 was isolated from a 2-3 L of budding yeast cell culture, following the ‘concentrated cell extract’ method described previously (Maric et al., 2014). After two hours of incubation with IgG-coated magnetic beads, the beads were washed 3 times with 1ml of TEV wash buffer (100mM Tris-acetate pH 9.0, 100mM potassium acetate, 10mM magnesium acetate, 0.1% (v/v) IGEPAL CA-630) and then once with 1ml of TEV cleavage buffer (100mM HEPES-KOH pH 7.9, 100mM potassium acetate, 10mM magnesium acetate, 0.1% (v/v) IGEPAL CA-630, 2mM EDTA-KOH). The beads were then incubated for 1 hour at 24°C in 50μl of TEV cleavage buffer containing 10 units of AcTEV protease (Thermo Scientific), with shaking at 1400 rpm on an Eppendorf Thermomixer F1.5. The eluted material was transferred to a fresh microfuge tube and 25 μl of 3X Laemmli buffer was added, before the sample was boiled at 95°C for 5 minutes and snap frozen.

#### Purification of recombinant proteins from *E. coli*

##### Cdc48 (wild-type and Cdc48-2A)

Cdc48 was expressed in *E. coli* and purified essentially as described previously (Bodnar and Rapoport, 2017b). The plasmids expressing 14His-Smt3-Cdc48 are listed in the Key Resources Table, and were transformed into BL21-CodonPlus (DE3)-RIL competent *E. coli* (230245, Agilent). Transformants were inoculated into a 200ml starter culture of LB medium containing 30 μg / ml each of kanamycin and chloramphenicol, before growth overnight at 37°C with shaking at 180 rpm. The following day, the culture was diluted to an OD_600_ of 0.15 in 1 L of LB medium containing with 30μg / ml each of kanamycin and chloramphenicol, and then grown at 30°C to an OD_600_ of 0.7. At this point, 0.5mM of IPTG was added to induce expression of Cdc48 for 2 hours. Cells were subsequently harvested and the pellet was washed once with cold PBS, before storage at −80°C.

Subsequently, the cell pellet was thawed and re-suspended in 20 mL of lysis buffer (50mM Tris-HCl pH 8.0, 500mM NaCl, 40mM Imidazole, 5mM Magnesium Acetate, 0.1mM ATP, 0.5mM TCEP, 1mM PMSF and 1.5mM pepstatin-A). To this suspension, 1mg/ml lysozyme (Sigma, cat. no. 10837059001) and 1μl of Pierce Universal Nuclease (Fisher, cat. no. PN88702) were added and the sample incubated at room temperature for 30 minutes with constant mixing on a rotating wheel. The cell debris was removed by centrifugation at 25000 g for 30 minutes, and the cleared lysate was subjected to Ni^2+^ affinity purification by incubation with 1 mL packed bead volume of Ni-NTA resin (QIAGEN) for 2 hours at 4°C, with constant mixing on a rotating wheel. The beads were recovered in a Poly-Prep chromatography column (Bio-Rad cat. no. 731-1550) and then washed extensively with 50 column volumes of lysis buffer.

Cdc48 was then eluted with six column volumes of elution buffer (50 mM Tris-HCl pH 8.0, 500 mM NaCl, 500 mM Imidazole, 5 mM Magnesium Acetate, 0.1 mM ATP, 0.5mM TCEP). The eluate was collected in 2 mL aliquots, and peak eluate fractions were then pooled and treated for 30 minutes at 4°C with 1 / 50 (v:v) aliquot of 1 mg / ml yeast Ulp1 SUMO protease (kind gift from Dr. Helen Walden), in order to cleave off the His_14_-SUMO tag from the amino terminus of Cdc48.

The Ulp1-cleaved eluate was concentrated down to 500μl using an ‘Amicon’ concentrator with a 10kDa cut-off filter (Amicon, cat. no. UFC901024), spinning at 21,000 x g for 15-20 minutes at 4°C. Untagged Cdc48 was then separated from Ulp1 and the 14His-Smt3 tag by gel filtration using the AKTA Pure protein purification system (GE Helathcare Lifesciences), with a 24 mL Superose 6 column (GE Healthcare Lifesciences) equilibrated with gel filtration buffer (20mM HEPES-KOH pH 7.6, 300mM Sorbitol, 150mM NaCl, 5mM Magnesium Acetate, 0.1mM ATP, 0.5mM TCEP). Fractions of 1 mL were collected and pooled peak fractions were concentrated to 500μl using a 10kDa cut-off spin filter.

Contaminants were removed by anion exchange chromatography using HiTrap Q (GE Healthcare Lifesciences) and the Akta Pure system. Protein was eluted with a 20 column-volume gradient from 0.15 to 1 M NaCl. Cdc48-containing fractions were pooled and dialysed against 20mM HEPES-KOH pH 7.6, 300mM Sorbitol, 150mM NaCl, 5mM Magnesium Acetate, 0.1mM ATP, 0.5mM TCEP for 4 hours at 4°C with stirring. The dialysed sample was recovered, aliquoted and snap frozen.

##### Shp1

A plasmid expressing 6His-Shp was transformed into BL21-CodonPlus (DE3)-RIL competent *E. coli* (230245, Agilent). The recombinant protein was then expressed and purified by Ni^2+^ affinity chromatography, as described above for Cdc48. The peak elution fractions were then aliquoted and snap frozen.

#### *In vitro* CMG ubiquitylation assay

A complex of SCF^Dia2^ with the yeast replisome was isolated as described above, and then released from beads by TEV cleavage in 50 μl *in vitro* ubiquitylation buffer (100mM HEPES-KOH pH 7.9, 100mM Potassium Acetate, 10 mM Magnesium Acetate, 0.02% (v/v) IGEPAL CA-630, 1mM DTT). The human deubiquitylating enzyme HsUSP2 (DU13025, MRC PPU Reagents and Services) was then added to 0.2 μM and incubated for 1 hour at 24°C, with agitation at 1400 rpm on an Eppendorf Thermomixer F1.5. Subsequently, HsUSP2 was inactivated for 15 minutes at 24°C by addition of 0.1 μM propargylated ubiquitin (kindly provided by Axel Knebel, Clare Johnson and MRC PPU Reagents and Services). Finally, ubiquitylation was induced by addition of 300 nM Uba1 E1 enzyme (kindly provided by Axel Knebel, Clare Johnson and MRC PPU Reagents and Services), 150 nM E2 enzyme Cdc34 (kindly provided by Tom Deegan), 0.5 μM human ubiquitin (kindly provided by Axel Knebel, Clare Johnson and MRC PPU Reagents and Services) and 5 mM ATP (Sigma, cat. no. GE27-2056-01), before incubation at 24°C for 30 minutes. The reactions were stopped by addition of 25 μl of 3X Laemmli buffer and then boiled for 5 minutes at 95°C before analysis by immunoblotting.

#### Reconstituted CMG disassembly assay

Ubiquitylated CMG (50 μl reaction as described above) was mixed with 50 nM Cdc48 and 50 nM of Ufd1-Npl4 heterodimer (kindly provided by Tom Deegan). The reaction was allowed to proceed at 24°C for 1 hour, after which 10 μl of slurry of magnetic beads couple of M2 anti-FLAG antibody (F3165, Sigma-Aldrich) was added, in order to isolate the CMG helicase via the FLAG-tagged Psf2 subunit of GINS. The immunoprecipitation was performed at 4°C for 1h, with constant mixing of beads on a rotating wheel. The supernatant was then combined with 25 μl of 3X Laemmli buffer, and 50 μl of 1X Laemmli buffer was added to the beads, before heating for 5 minutes at 95°C. The eluates were then snap frozen before subsequent analysis by SDS-PAGE and immunoblotting (using equivalent proportions of bead and supernatant fractions).

#### Primary antibodies

Sheep polyclonal antibodies to Mcm2-3-5-6-7, Cdc45, Psf1-2-3, Sld5, Ctf4, Csm3, Mrc1, Cdc48, Ufd1 and Npl4 were all described previously (Maric et al., 2014), and are summarized in the Key Resources Table. Polyubiquitin was detected with FK2 monoclonal antibody (Enzo Life Sciences, BML-PW8810) and TAP or ProteinA-tagged proteins were detected with Phosphatase-anti-phosphatase complex (PAP; Sigma, P1291).

#### Secondary antibodies

The following secondary antibodies were used: anti-sheep HRP (Sigma, A3415); anti-rabbit HRP (GE Healthcare, NA9340); anti-mouse HRP (Vector Labs, PI-2000).

### Quantification and Statistical Analysis

This study does not generate quantitative data that requires statistical analysis.

### Data and Code Availability

This study did not generate or analyze datasets or code.

## Supplemental Information

Document S1. Figures S1–S4 and Table S1

Document S2. Article plus Supplemental Information
