# Supplementary material for: In Vitro Reconstitution Defines the Minimal Requirements for Cdc48-Dependent Disassembly of the CMG Helicase in Budding Yeast
Source: Cell Rep. 2019 Sep 10;28(11):2777–2783.e4. doi: 10.1016/j.celrep.2019.08.026 (PMC6899518; doi:10.1016/j.celrep.2019.08.026)
Supplement: Document S1. Figures S1–S4 and Table S1 [file mmc1.pdf]

Cell Reports, Volume *28*

Supplemental Information

In Vitro Reconstitution Defines the Minimal Requirements for Cdc48-Dependent Disassembly of the CMG Helicase in Budding Yeast

Progya P. Mukherjee and Karim P.M. Labib

Figure S1, related to Figures 1-4

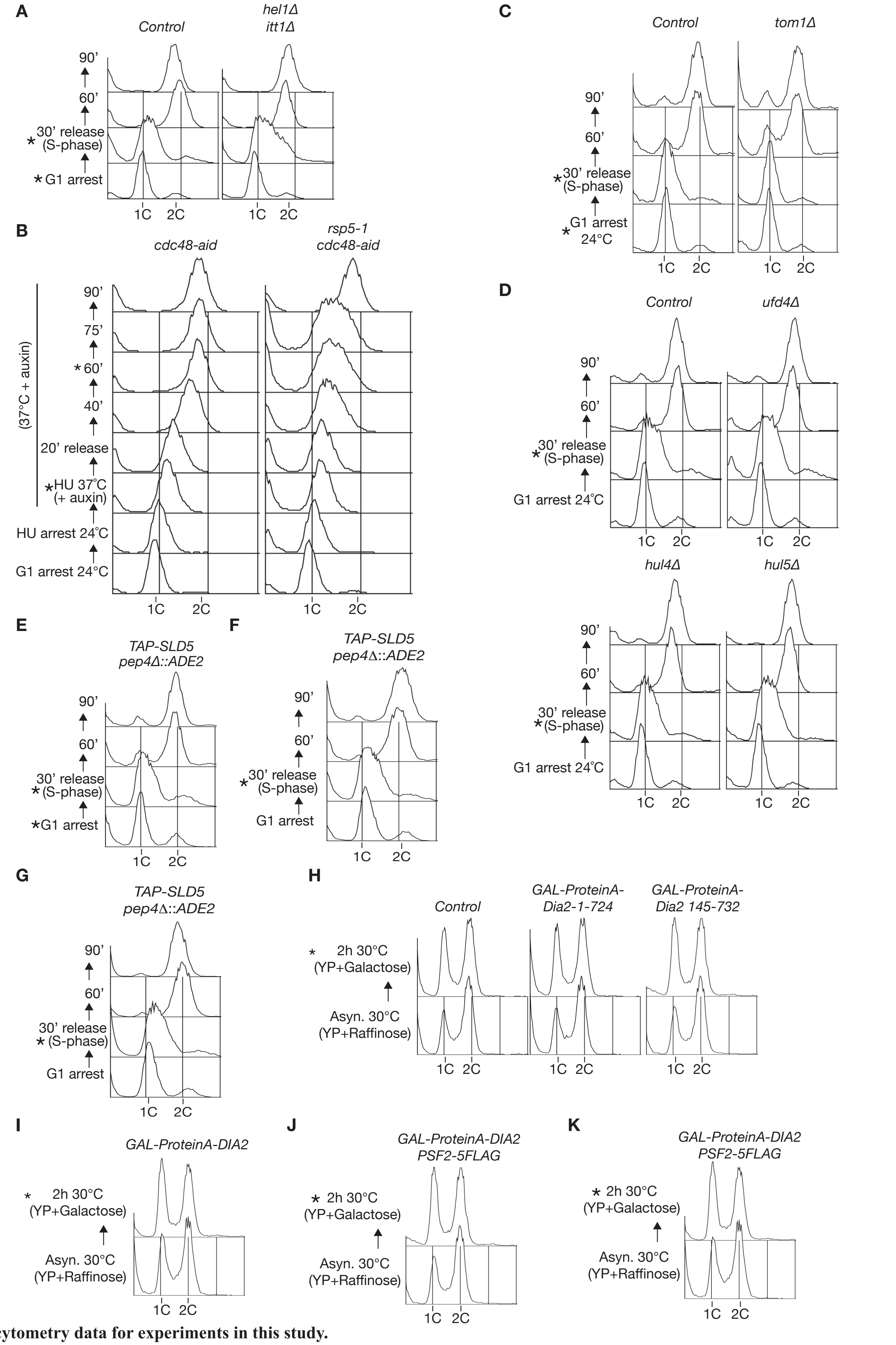

Flow cytometry data for experiments in this study.

The figure presents flow cytometry data for the experiments in Figure 1A-D (**A**-**D**), Figure 2A-C (**E**-**G**), Figure 4A-B (**H**-**I**), Figure 4B-C (**J**-**K**). The samples marked with asterisks were used to prepare cell extracts.

Figure S2, related to Figures 1-2

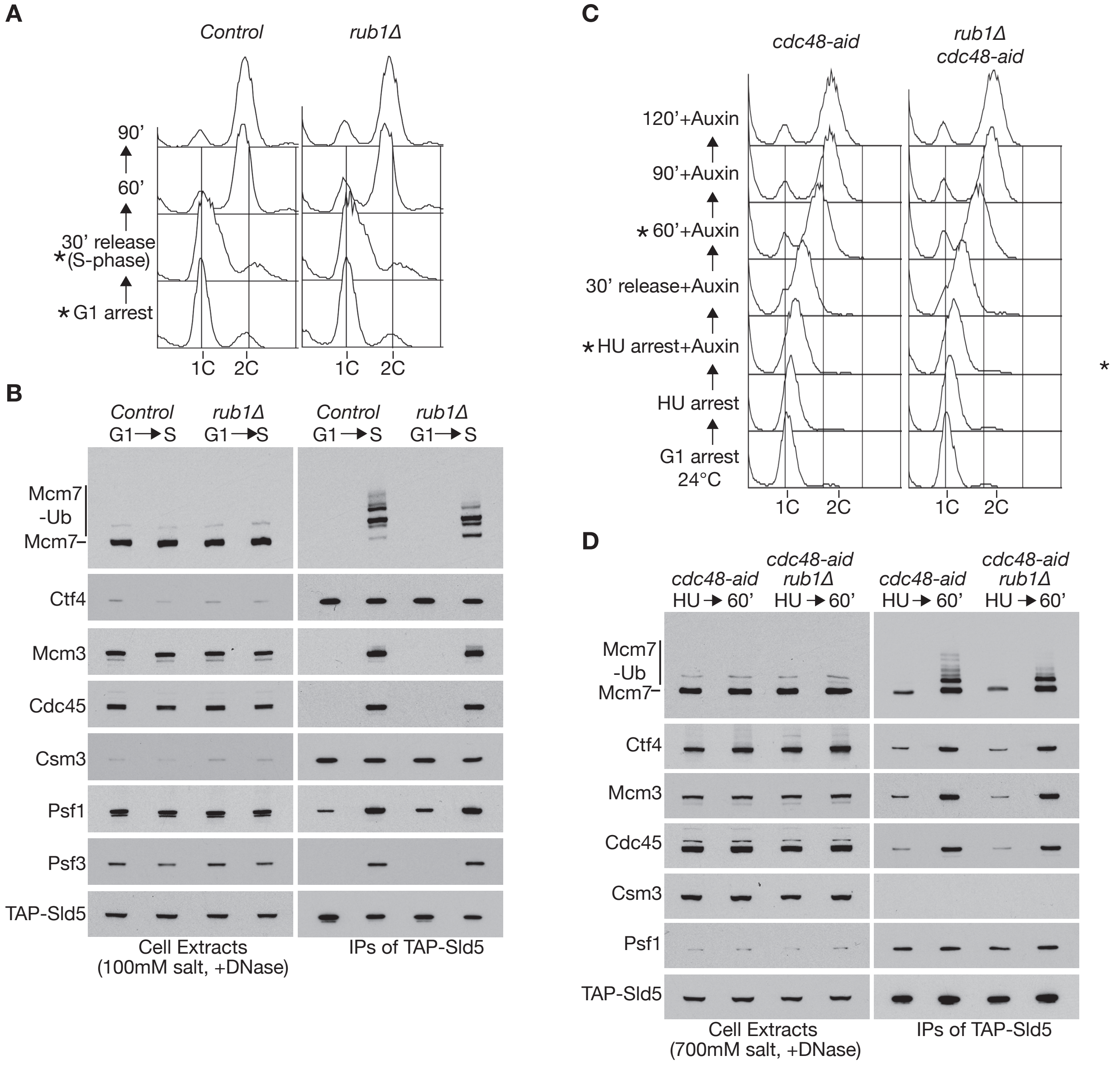

Cullin neddylation is not essential for CMG helicase ubiquitylation in budding yeast.
(**A**) Control (YSS47) and *rub1*Δ (YPM35) cells were grown at 24˚C, synchronised in G1 phase by addition of mating pheromone, and then released into S phase. Samples were taken at the indicated times and processed for flow-cytometry. The samples marked with asterisks were used to make cell extracts. (**B**) Samples from the same experiments were used to monitor CMG helicase ubiquitylation in yeast cell extracts, as in Figure 1A. (**C**) *cdc48-aid* control cells (YMM228) and *cdc48-aid rub1*Δ cells (YPM29) were grown at 24˚C, synchronised in G1 phase by addition of mating pheromone, and then released into medium containing 0.2 M hydroxyurea (HU) at 24˚C, until more than 80% of the cells had budded. The auxin indoleacetic acid was then added to 500 µM, in order to deplete Cdc48-aid. The cultures were subsequently released into fresh medium containing auxin but lacking HU, so that cells were able to complete S phase. Samples were taken at the indicated times and processed for flow-cytometry. The samples marked with asterisks were also used to prepare 'high-salt' cell extracts (containing 700mM potassium acetate), in order to monitor *in vivo* CMG ubiquitylation in the absence of in vitro ubiquitylation. (**D**) TAP-Sld5 was isolated by immunoprecipitation from extracts of the indicated samples from (C).

Figure S3, related to Figures 3-4

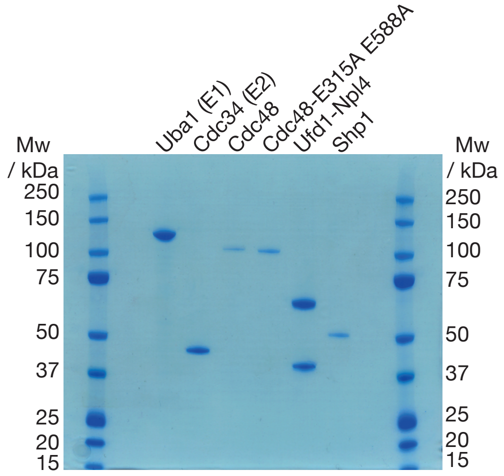

Purified proteins employed in this study.
The indicated factors were expressed in recombinant form and purified as described in Methods, before resolution in a 4-12% Bis-Tris gel, and staining with colloidal Coomassie blue.

Figure S4, related to Figure 3

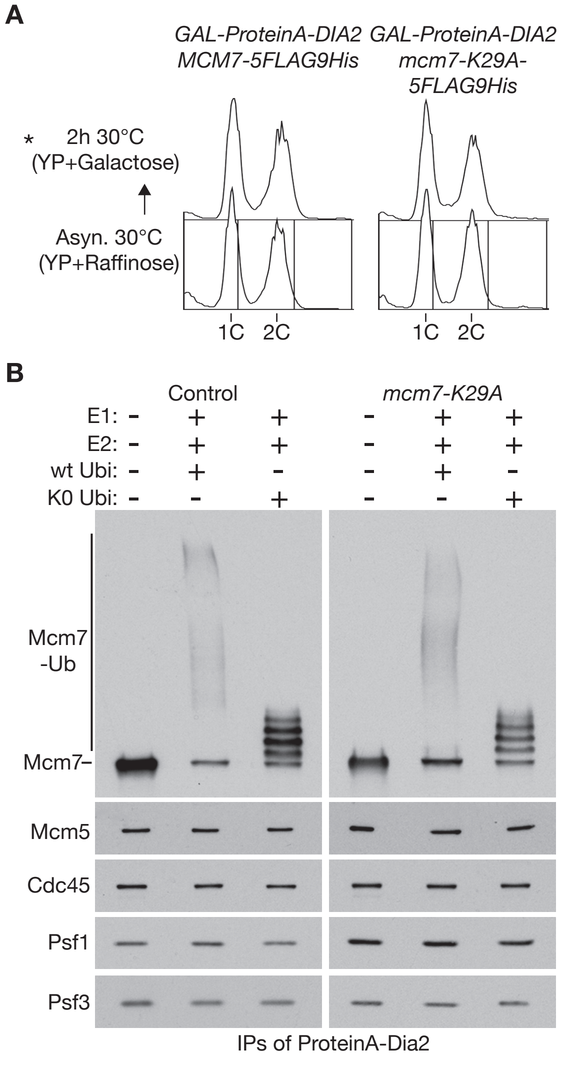

***In vitro* ubiquitylation of the CMG helicase within isolated complexes of SCF^{Dia2}-replisome is not restricted to lysine 29 of Mcm7.** (**A**) Strains expressing *GAL-ProteinA-TEV-DIA2 MCM7-5FLAG9His* (YPM182) or *GAL-ProteinA-TEV-DIA2 mcm7-K29A-5FLAG9His* (YPM164), were grown at 30˚C in raffinose-containing media until mid-exponential phase. The ProteinA-tagged versions of Dia2 were then induced by addition of galactose for 2 hours. Samples was taken at the indicated times and processed for flow-cytometry. The samples marked with asterisks were also used to prepare cell extracts. (**B**) ProteinA-tagged Dia2 was then isolated and processed as in Figure 3B, either using 50 μM wild type ubiquitin (wt Ubi) or 50 μM lysine-free ubiquitin (K0 Ubi), before immunoblotting of the indicated factors.

Strain	Genotype
W303-1	*MAT***a** *ade2-1 ura3-1 his3-11,15 trp1-1 leu2-3,112 can1-100 /* *MAT*α *ade2-1 ura3-1 his3-11,15 trp1-1 leu2-3,112 can1-100*
W303-1a	*MAT***a** *ade2-1 ura3-1 his3-11,15 trp1-1 leu2-3,112 can1-100*
YSS47	*MAT***a** *TAP-SLD5 (kanMX) pep4Δ::ADE2*
YTM418	*MAT***a** *ura3::GAL-ProteinA-3TEV-DIA2 pep4Δ::ADE2*
YTM495	*MAT***a** *ura3::GAL-ProteinA-3TEV-Dia2-(145-732) pep4Δ::ADE2*
YMM228	*MAT***a** *TAP-SLD5 (kanMX) cdc48-aid (hphNT) ADH1-O.s.TIR1-9MYC (URA3 & K.l.TRP1) ADE2 pep4Δ::URA3*
YSS3	*MAT***a** *pep4Δ::ADE2*
YPM29	*MAT***a** *rub1Δ::K.l.TRP1 TAP-SLD5 (kanMX) cdc48-aid (hphNT) ADH-O.s.TIR1-9MYC (URA3) pep4Δ::ADE2*
YPM35	*MAT***a** *rub1Δ::K.l.TRP1 TAP-SLD5 pep4Δ::ADE2*
YPM44	*MAT***a** *tom1Δ::URA3 TAP-SLD5 pep4Δ::ADE2*
YPM106	*MAT***a** *hel1Δ::K.l.TRP1 TAP-SLD5 (kanMX) pep4Δ::ADE2*
YPM122	*MAT***a** *itt1Δ::URA3 TAP-SLD5 (kanMX) pep4Δ::ADE2*
YPM141	*MAT***a** *hel1Δ::K.l.TRP1 itt1Δ::URA3 TAP-SLD5 (kanMX) pep4Δ::ADE2*
YPM150	*MAT***a** *ufd4Δ::URA3 TAP-SLD5 (kanMX) pep4Δ::ADE2*
YPM153	*MAT***a** *rsp5-1 TAP-SLD5 (kanMX) pep4Δ::ADE2*
YPM157	*MAT***a** *ura3::GAL-ProteinA-3TEV-DIA2 PSF2-5FLAG (hphNT) pep4Δ::ADE2*

YPM164	*MAT***a** *ura3::GAL-ProteinA-3TEV-DIA2 (URA3) mcm7-K29A-5FLAG9His (hphNT) pep4Δ::ADE2*
YPM174	*MAT***a** *rsp5-1 TAP-SLD5 (kanMX) cdc48-aid (hphNT) ADH1-O.s.TIR1 (URA3 & K.l.TRP1) pep4Δ::ADE2*
YPM182	*MAT***a** *ura3::GAL-PrA-3TEV-DIA2 MCM7-5FLAG9HIS (hphNT) pep4Δ::ADE2*
YPM192	*MAT***a** *hul4Δ::K.l.TRP1 TAP-SLD5 (kanMX) pep4Δ::ADE2*
YPM195	*MAT***a** *hul5Δ::HIS3 TAP-SLD5 (kanMX) pep4Δ::ADE2*
YPM220	*MAT***a** *pRS306-ProteinA-3TEV-dia2-(1-724) pep4Δ::ADE2*

Table S1, Related to Figures 1-4.
Strains used in this study (all based on the W303 strain background).
